# Wet-Chemical Synthesis of 3D Stacked Thin Film Metal-Oxides for All-Solid-State Li-Ion Batteries

**DOI:** 10.3390/ma10091072

**Published:** 2017-09-12

**Authors:** Evert Jonathan van den Ham, Giulia Maino, Gilles Bonneux, Wouter Marchal, Ken Elen, Sven Gielis, Felix Mattelaer, Christophe Detavernier, Peter H. L. Notten, Marlies K. Van Bael, An Hardy

**Affiliations:** 1 Inorganic and Physical Chemistry and Imec, Division Imomec, Institute for Materials Research, Hasselt University, Martelarenlaan 42, 3500 Hasselt, Belgium; jonathan.vandenham@uhasselt.be (E.J.v.d.H.); giulia.maino@uhasselt.be (G.M.); gilles.bonneux@uhasselt.be (G.B.); wouter.marchal@uhasselt.be (W.M.); Ken.elen@uhasselt.be (K.E.); gielissven@hotmail.com (S.G.); marlies.vanbael@uhasselt.be (M.K.V.B.); 2 Imec vzw, Division Imomec, Wetenschapspark 1, B-3590 Diepenbeek, Belgium; 3 Department of Solid State Sciences, Ghent University, Krijgslaan 281 S1, 9000 Gent, Belgium; felix.mattelaer@ugent.be (F.M.); christophe.detavernier@ugent.be (C.D.); 4 Energy Materials & Devices, Eindhoven University of Technology, 5600 MB Eindhoven, The Netherlands; p.h.l.notten@tue.nl; 5 Forschungszentrum Jülich, Fundamental Electrochemistry (IEK-9), D-52425 Jülich, Germany

**Keywords:** ultrasonic spray deposition, tungsten oxide, lithium lanthanum titanium oxide, conformal coating, Li-ion batteries

## Abstract

By ultrasonic spray deposition of precursors, conformal deposition on 3D surfaces of tungsten oxide (WO_3_) negative electrode and amorphous lithium lanthanum titanium oxide (LLT) solid-electrolyte has been achieved as well as an all-solid-state half-cell. Electrochemical activity was achieved of the WO_3_ layers, annealed at temperatures of 500 °C. Galvanostatic measurements show a volumetric capacity (415 mAh·cm^−3^) of the deposited electrode material. In addition, electrochemical activity was shown for half-cells, created by coating WO_3_ with LLT as the solid-state electrolyte. The electron blocking properties of the LLT solid-electrolyte was shown by ferrocene reduction. 3D depositions were done on various micro-sized Si template structures, showing fully covering coatings of both WO_3_ and LLT. Finally, the thermal budget required for WO_3_ layer deposition was minimized, which enabled attaining active WO_3_ on 3D TiN/Si micro-cylinders. A 2.6-fold capacity increase for the 3D-structured WO_3_ was shown, with the same current density per coated area.

## 1. Introduction

Finding smart solutions for sustainable energy harvesting and storage is often opted as the main challenge of the near future. Lithium ion (Li-ion) batteries are major candidates for energy storage due to their superior energy and power density compared to other battery technologies. However, contemporary Li-ion batteries suffer from a number of intrinsic issues, mostly related to the use of liquid electrolyte: (i) safety risks; (ii) limited lifetime; and (iii) operating temperature limitations. Efforts are made to tackle these issues by stabilizing polymer and gel-based electrolytes, in combination with contemporary battery design, increasing the thermal stability and lifetime of the battery [1,2]. However, these issues can be dealt with to a greater extent by adopting a solid-electrolyte, yielding an all-solid-state battery [3,4]. However, all-solid-state batteries suffer from intrinsic issues as well, due to the lower conductivity of the solid-electrolyte. To avoid internal resistance, resulting in an ohmic drop, a thin film approach is often adopted [5]. Although this yields rate capability enhancements, it comes at a cost: thin film electrodes are intrinsically limited in capacity due to their limited volume. Simply thickening the electrodes would lead to high resistance as well, as Li-ions and electrons have to diffuse through a thicker layer. Therefore, the concept of 3D all-solid-state batteries was proposed [6]. More specifically, the combination between the thin-film approach and 3D batteries was proposed by Notten et al. leading to the integrated 3D all-solid-state Li-ion battery [7].

To achieve this challenging goal, several positive and negative electrode materials (often referred to as cathodes and anodes, respectively) have been studied for 3D deposition using high aspect ratio scaffolds [7]. For electrode materials, conformal coatings of Li_4_Ti_5_O_12_ [8], LiCoO_2_ [9,10], and (Li)FePO_4_ were shown before [11], leading to an increased capacity due to the use of 3D geometries [6]. For solid-electrolytes, reports were found on the deposition of LiPO(N), LiTaO_3_ and lithium silicates [12,13,14,15,16,17]. Recently, stacked electrodes and solid-electrolytes were shown, creating an all-solid-state 3D Li-ion battery [18,19]. In all these cases, vacuum based methods were required. Despite good performance of these materials, from an upscaling perspective, this is a costly route. In addition, the choice of materials is dominated by specific diffusion behavior of gas precursors used. In this study, an alternative is presented: conformal coatings of oxide materials for Li-ion batteries using ultrasonic spray deposition. Based on a breakthrough reported previously regarding non-planar deposition using wet-chemical methods [20,21], 3D all-solid-state Li-ion batteries could be within reach at much lower costs and a wider choice of oxide materials. Based on previous results using this approach [21], this study aims to investigate the stacking of tungsten oxide (WO_3_) as a negative electrode in combination with amorphous lithium lanthanum titanium oxide (Li_3x_La_(2/3)−x_TiO_3_, referred to as LLT) solid-electrolyte to compile a 3D all-solid-state half-cell.

Although WO_3_ is mostly known within the field of electrochromic devices [22,23,24,25,26,27,28], and photovoltaics [29], it also serves as a negative electrode material for Li-ion batteries [22,30,31,32,33]. Though the gravimetric capacity of this electrode material is mediocre, the volumetric energy density is higher than most other oxide electrode materials. For instance, the volumetric density of WO_3_ is 604 mAh·cm^−3^ [33,34], whereas the Li_4_Ti_5_O_12_ (LTO) has a capacity of only 228 mAh·cm^−3^ [5]. For all-solid-state batteries the latter is arguably the most important device parameter. Furthermore, as is the case for LTO, WO_3_ only shows a small volume change during lithiation [35]. The volume change proved to yield no practical issues for thin films up to 100 cycles [33]. In addition, the choice for WO_3_ is related to the fact that the structure can crystallize at temperatures below 300 °C [24,36], which makes it interesting from a device integration point view. Finally, WO_3_ exhibits an intrinsic advantage related to the compatibility with the solid-electrolyte material. With a relative high (de)intercalation voltage for a negative electrode material—ranging from 1.5 to 3 V vs. Li/Li^+^ [22,29,30,33]—WO_3_ is compatible with the electrochemically relatively unstable LLT electrolyte [33]. The latter material suffers from a Ti^4+^ reduction while cycling below 1.5 V vs. Li^+^/Li, making it unsuitable for many negative electrode materials such as lithium and graphite [3]. In fact, all Ti-based electrolytes are susceptible to this problem, meaning that various materials NASICON electrolyte class suffers from this reduction as well [37,38]. Indeed, more stable solid-electrolytes—compatible with metallic lithium—are available such as “Li-stuffed” garnets [39,40,41]. However, besides vacuum based deposition of LiPO(N) and LiTaO_3_ [12,13,14,15,17], no 3D compatible deposition methods have been established for these stable electrolyte materials.

Notably, the choice was made to prepare LLT in its amorphous state. Previous studies indicated that crystallization of the highly Li^+^ conductive perovskite lithium lanthanum titanium solid-electrolyte leads to serious issues regarding cracks and pinholes [42,43]. Therefore, at the cost of a lower Li-ion conductivity of 10^−8^ S·cm^−1^, the amorphous form of this material is chosen. This leads to enhanced morphology, which is of crucial importance to prevent short circuits over the electrolyte layer [43]. In addition, the more mild annealing conditions required for the amorphous phase eases integration with other materials, which is crucial for 3D all-solid-state Li-ion batteries.

## 2. Materials and Methods

### 2.1. Synthesis of the Tungsten Precursor (W-Precursor)

A W-precursor solution was prepared by adding tungstic ac id (H_2_WO_4_, ≥99%, Sigma Aldrich, Overijse, Belgium) and citric acid hydrate (CA, ≥99%, Sigma Aldrich, Overijse, Belgium) to a round bottom flask, dispersed with a small amount of water. The H_2_WO_4_ to CA ratio was 1:4. This yellow suspension was stirred and heated at 120 °C for 2 h under reflux conditions. Subsequently, the pH was raised to pH > 12 with ammonia (NH_3_, 32%, Merck, Overijse, Belgium) and left to stir for 24 h. After cooling, a transparent, grey colored solution was obtained. The final pH was 8, with a concentration of 0.35 mol·L^−1^, as was determined by inductively coupled plasma-atomic emission spectroscopy (ICP-AES, Optima 3300, PerkinElmer, Zaventem, Belgium). Before ultrasonic spray deposition, the precursor was diluted and mixed with ethanol (10:9 water/ethanol volume ratio) to yield a 25 mM concentration [20,21].

### 2.2. Synthesis of the Lithium Lanthum Titanium Precursor (Li-La-Ti Precursor)

The Li-La-Ti-precursor used was reported earlier based on work in our labs [43]. The citrate-nitrate precursor was combined with ethanol (10:9 water/ethanol volume ratio) with a final (total) metal-ion concentration of 10 mM.

### 2.3. Substrates

Three different types of substrates were used for this study: (i) Pt (sputtered) and TiN (CVD)-coated silicon wafer; (ii) trench-structured Si wafer, prepared by reactive ion etching (Philips, Amsterdam, The Netherlands); and (iii) TiN coated silicon micro-cylinders, prepared by reactive ion etching (IMEC, Leuven, Belgium). All these substrates were cleaned by a UV/O_3_ treatment at 60 °C (30 min, PSD Pro Series, Novascan, Ames, IA, USA) prior to deposition.

### 2.4. Thin Film Synthesis

The precursors were deposited via ultrasonic spray deposition (Exacta Coat, Sono-Tek Cooperation, Milton, NY, USA) with a deposition temperature set at 180 °C for the tungsten precursor and 200 °C for the Li-La-Ti-precursor. The liquid was dispensed at 0.2 mL·min^−1^ and the carrier gas (N_2_) pressure was set at 1.5 psi. The spray nozzle to the substrate distance was 2.7 cm. The nozzle moved with a speed of 100 mm·s^−1^. The number of deposition cycles was varied (2 to 20) with 5 s dry time between the deposition cycles, including intermediate heat treatments every 5 cycles. The deposited W-precursor was annealed at 600 °C for 1 h on the Pt planar substrate, 10 min at 500 °C for 10 min on the other substrates. Deposited Li-La-Ti-precursor was annealed at 500 °C for 1 h on a hotplate for Pt planar substrates, 10 min at 500 °C for on the other substrates.

### 2.5. Characterization

The thermal decomposition profile of the dried W- and Li-La-Ti-precursor gel, obtained by drying the precursor solution at 60 °C, was investigated by thermogravimetric analysis with coupled differential scanning calorimetry (TGA-DSC, SDT Q600, TA instruments, Asse, Belgium). Six milligrams of the gel was heated at 10 °C·min^−1^ using dry air (0.1 L·min^−1^) in an alumina crucible. The thermal decomposition profile of the W-precursor films was investigated by thermogravimetric analysis coupled with mass spectrometry (TG-MS, Q5000 with Pfeiffer quadrupole MS, Asse, Belgium). The film was obtained by W-precursor deposition on thin borosilicate glass (Micro Cover Glasses, thickness 0.08 mm, VWR, Oud-Heverlee, Belgium). The coated glass was heated in a Pt sample crucible, ramped at 10 °C·s^−1^ from room temperature to 500 °C in static air.

Crystallization of the WO_3_ films deposited on Pt was investigated by XRD via a Bruker D8 equipped with a linear strip detector, using a step size of 0.040° and a counting time of 0.3 s per step at room temperature. Crystallization of non-annealed W-precursor films on TiN coated Si micro-cylinders, was studied by in-situ heating XRD (D8 Discover, Bruker, Champs-sur-Marne, France) with experimental heating chamber [44]. The morphology and film thicknesses were investigated via scanning electron microscopy (SEM, Quanta 200F, FEI, Zavetem, Belgium), cross-sections were made by cutting the substrates with a diamond pen. In addition, the average film thickness of WO_3_ films was determined by ICP-AES, after dissolving the deposited films in NH_3_ (overnight). Cyclic voltammetry and galvanostatic measurements were done with an Autolab PGSTAT128N, using a three-electrode setup with a custom made Teflon cell similar to the setup used before [10]. The counter and reference electrodes consisted of metallic lithium (99.9% Sigma Aldrich, Overijsse, Belgium). The measurements were done in 1.0 M LiClO_4_ in propylene carbonate (Soulbrain, Northville, MI, USA). After 3 cycles of CV at 1 mV·s^−1^ between 2 and 3.5 V, galvanostatic measurements were done with the same cut-off voltages with various (dis)charge currents. The test cell was operated at 20.0 °C in a control chamber, inside an Ar-filled glovebox with H_2_O and O_2_ concentrations < 1 ppm. In addition, cyclic voltammetry was done at 10 mV·s^−1^ for 5 cycles in 0.1 M of ferrocene (Sigma Aldrich, ≥98%, Overijsse, Belgium) in anhydrous acetonitrile (ATN; ≥99.9%, VWR, Oud-Heverlee, Belgium), using a Ag/AgNO_3_ (0.01 M) in 0.1 M Bu_4_NPF_6_ as reference electrode, and acetonitrile with 0.1 M Bu_4_NPF_6_ as counter electrode. The ferrocene solution was bubbled in N_2_ for 2 to 3 min before starting of the experiment.

## 3. Results and Discussion

### 3.1. Planar Tungsten Oxide and Lithium Lanthanum Titanium Oxide Stacks

#### 3.1.1. Thermal Decomposition of the Precursor

**Figure 1:**
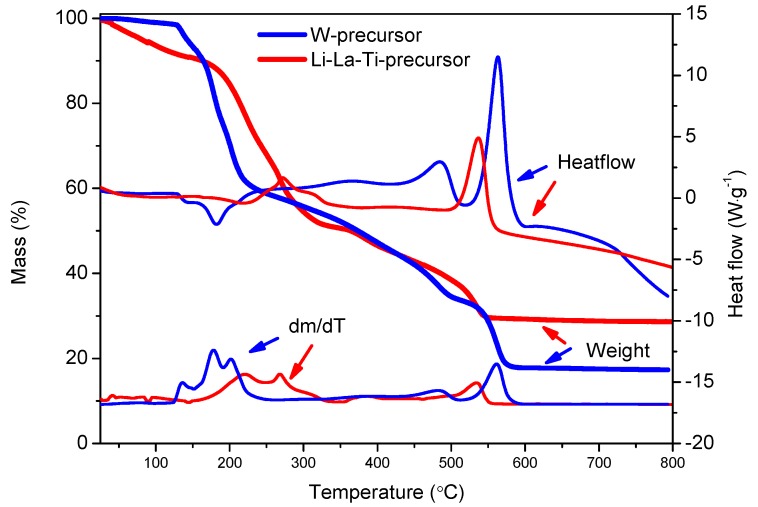
TGA-DSC analysis of the W- and Li-La-Ti-precursors dried at 60 °C, recorded at 10 °C·min^−1^ in dry air.

The decomposition of the W-precursor is shown in Figure 1. After evaporation of residual water from the precursor gel due to incomplete drying, a first weight loss is observed at 140 and 180 °C with endothermal features. This is followed by two minor exothermal decomposition steps at 365 and 485 °C. Finally, a last major weight loss is observed at 560 °C during a strongly exothermic reaction. No significant weight loss is observed at higher temperatures, implying that the precursor is fully decomposed at 595 °C. Since the tungsten precursor was prepared with pH 8, the solution consists of tungsten complexes with three oxo-groups, one aqua group and a single citrate ligand coordinated to the tungsten, where the citrate co-exists in protonated and deprotonated form [45]. Since an excess of citric acid was initially required to completely dissolve the tungstic acid (4:1 citric acid to tungsten ratio), the excess of citric acid is converted to ammonium citrate due to the addition of ammonia. The presence of ammonium citrate can most clearly be observed during the endothermic peaks at 140 and 185 °C, ascribed to melting and decarboxylation reactions of the ammonium citrate, respectively [46]. Other features of the decomposition profile resemble decompositions of metal-citrate complexes studied before [47,48], although the temperature at which these reactions occur are shifted for this tungsten based complex, due to specific tungsten-oxygen interactions.

Since the Li-La-Ti-precursor is based on a different composition (citrates and nitrates) [43], this precursor decomposition shows different features than the W-precursor (solely citrates). First, the first major weight loss occurs at slightly higher temperatures, during a endothermic reaction at 220 °C, immediately followed by an exothermic reaction at 270 °C. Next, weight loss occurring after a small exothermal event at 420 °C continues towards the final major weight loss accompanied by strong heat generation at 535 °C. While comparing the separate decomposition profiles of the nitrate based-precursors (Figure S1) and titanium-citrato-peroxo precursor with small amounts of ammonium citrate [49], it becomes clear that the final decomposition temperature of the Li-La-Ti-precursor is lower than the separate starting products. Citrates and nitrates readily interact, forming a fuel-oxidizer mixture known from propellant chemistry [50]. Although the exact mechanism goes beyond the scope of this work, the interactions between citrates and nitrates at temperatures up to 250 °C may prevent formation of high temperature-stable lithium and lanthanum carbonates [51], yielding a lower final decomposition temperature.

#### 3.1.2. Morphology and Thickness of Deposited Films

**Figure 2:**
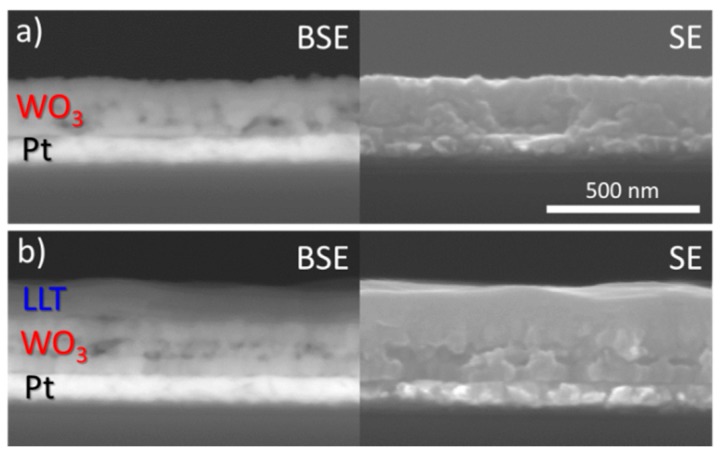
Cross-section SEM micrograph showing backscattered (BSE) and secondary electron (SE) images: (**a**) 10 cycles of W-precursor deposition on a planar Pt substrate, annealed at 600 °C; and (**b**) 20 cycles of Li-La-Ti-precursor deposition on WO_3_/Pt substrate, annealed at 500 °C.

Regarding the morphology, ultrasonic spray deposition of W-precursor yields 250 to 300 nm thick films, which look rather smooth, although porous features are visible (Figure 2a). The backscattering image shows a clear difference between the Pt and WO_3_. ICP-AES analysis of dissolved films show that an average thickness of 280 nm is obtained for 10 deposition cycles with the 25 mM W-precursor (27–28 nm/cycle). Upon deposition of the LLT on top, a slightly darker layer of 120 nm can be found on top of the WO_3_ (Figure 2b). To ensure a dense morphology, a lower precursor concentration was chosen to lower the growth rate (6 nm/cycle). The resulting LLT has a very smooth and dense morphology, due to the fact that the material remains amorphous at 500 °C [43]. Further heating towards the crystalline perovskite phase was not attempted, since previous attempts regarding a spin-coated film led to crack formation [43].

#### 3.1.3. Functional Properties of Planar Films—Electrochemical Activity

**Figure 3:**
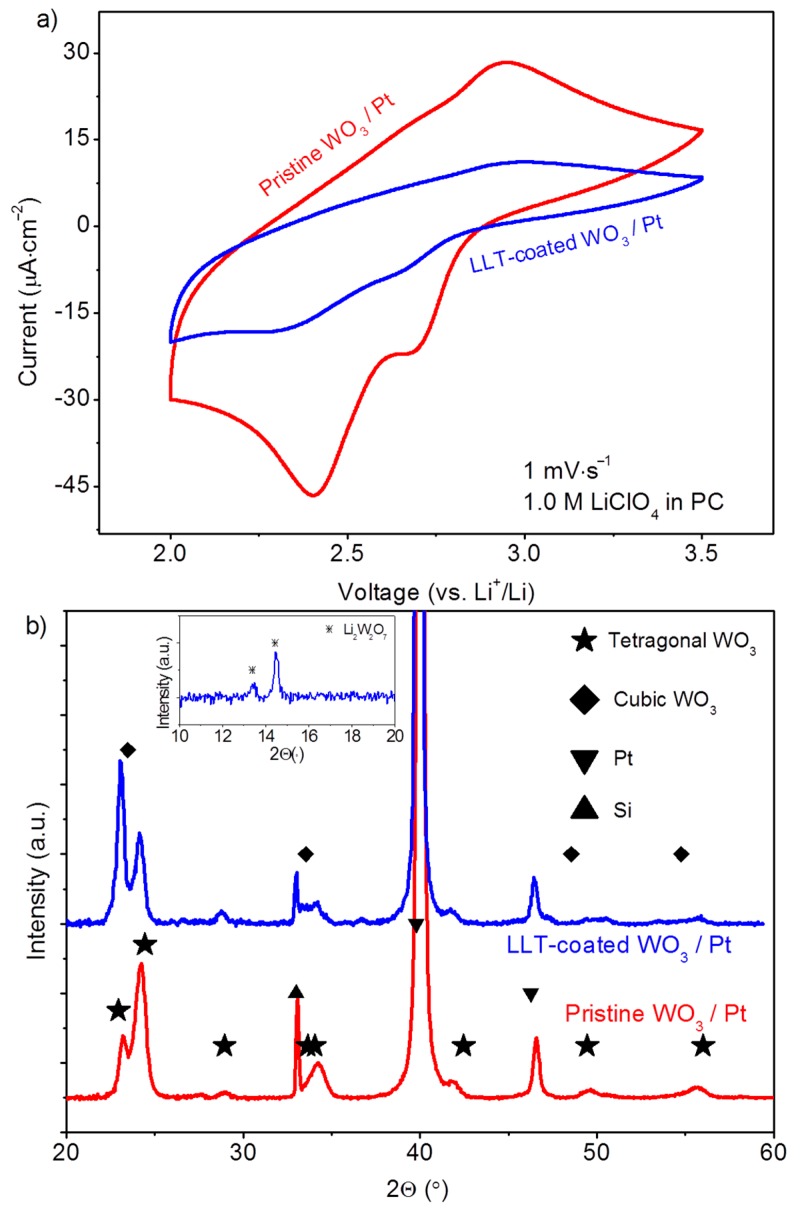
WO_3_-coated planar Pt substrate, annealed at 600 °C for 1 h in static air (red) and LLT-coated WO_3_ on a planar Pt substrate (prepared under the same conditions) annealed at 500 °C for 1 h in static air (blue), showing: (**a**) CV of the second cycles; and (**b**) XRD. Tetragonal WO_3_ (JCPDS 1-85-807), cubic WO_3_ (JCPDS 20-1323), Pt (JCPDS 1-87-647), Si (JCPDS 1-72-1088) and Li_2_W2O_7_ (JCPDS 28-598) phases are all indicated.

Electrochemical activity of the WO_3_ films as function of annealing temperature was probed by means of cyclic voltammetry, indicating that at least 400–600 °C annealing is required to obtain active films (not shown). With a 600 °C anneal, the films appeared to be most active (Figure 3a). Two distinct reduction peaks can be observed while cycling to lower potentials, associated with W^6+^ to W^5+^ (2.71 V) and W^5+^ to W^4+^ (2.40 V) reductions [52]. Although less intense, the oxidation reactions occur at 2.67 V (W^4+^ to W^5+^) 2.95 V (W^5+^ to W^6+^). The relative large currents at low voltages indicate kinetic hindrance effects due to mediocre Li^+^ and/or e^−^ conductivity. In principle, degenerate energy levels of amorphous WO_3_ phases could also be the cause of this [53], although the XRD analysis of the phase clearly indicates that significant amounts of crystalline WO_3_ are present (Figure 3b).

Upon coating the WO_3_ with LLT, the XRD analysis (Figure 3b) indicates that predominantly tetragonal WO_3_ remains present after the LLT coating. As the LLT is amorphous after the 500 °C anneal [43], no additional peaks were expected for this material. However, a close analysis reveals that the intensities of the peaks located at 23 and 24° (2θ) are reversed. Since the cubic phase of WO_3_ can be formed upon lithiation of WO_3_ [33], this change in intensity suggests a mixture of cubic and tetragonal WO_3_ is present in the LLT coated sample. The occurrence of this phase change is assigned to a reaction between Li^+^ and WO_3_. This is confirmed by additional analysis of the lower diffraction angles, revealing that minor amounts of Li_2_W_2_O_7_ are present due to a solid-state reaction as a result of lithium diffusion from the LLT layer to the WO_3_ underneath. Although Li_2_W_2_O_7_ is known as an electrode material with a larger storage capacity than WO_3_ [54], the intercalation voltage (1.55 V vs. Li^+^/Li) is too low to be compatible with the LLT electrolyte, which suffers from a Ti^4+^ reduction at lower voltages [3]. Hence, for this study, the Li_2_W_2_O_7_ is regarded as an undesirable but unavoidable secondary phase for the LLT-WO_3_ half-cell. With respect to the electrochemical activity, the current measured in the CV (Figure 3a) for the LLT-WO_3_ stack drops as compared to pristine WO_3_. In addition, a larger over-potential (approximately 50 mV) is noted in case of the LLT-coated WO_3_ sample. Although passivating effects of the electrolyte deposition and anneal are not excluded, possibly related to the formation of the Li_2_W_2_O_7_ phase, both the drop in current and the increase in over-potential can be related to the resistance induced by the amorphous solid-electrolyte layer on top of the WO_3_. Since amorphous LLT was reported to have a relatively low Li-ion conductivity (10^−8^ S·cm^−1^) using comparable synthesis conditions [43], these features are not surprising, but merely serve as a proof of the limited Li-ion conductivity of the electrolyte layer.

**Figure 4:**
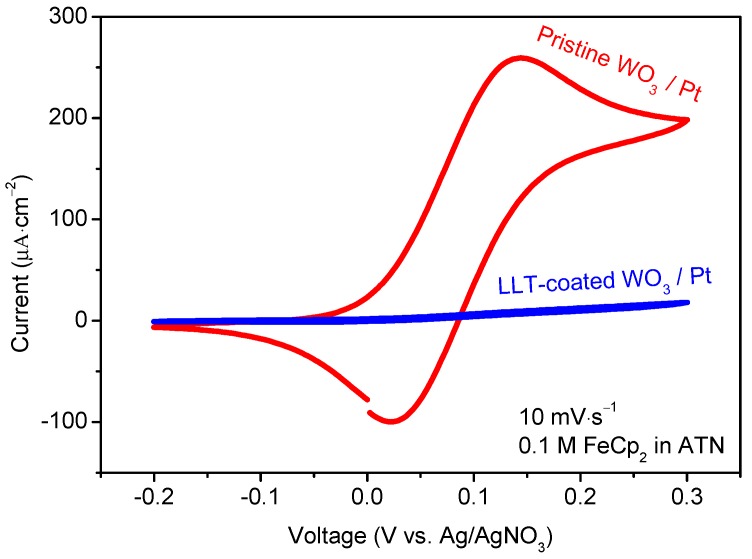
CV showing ferrocene reduction for 10 deposition cycles W-precursor deposition on a planar Pt substrate, annealed at 600 °C for 1 h in static air (red) and LLT-coated WO_3_ (prepared under the same conditions) annealed at 500 °C for 1 h in static air (blue). The samples were measured at 10 mV·s^−1^. The third cycles are shown.

Additional experiments were carried out to check the electron-blocking properties of the deposited electrolyte [16]. Figure 4 shows a CV of pristine and LLT-coated samples in the presence of ferrocene in a non-aqueous liquid electrolyte. Upon lowering the potential, ferrocene is reduced to ferrocenium because of electron-transfer at the surface of the sample. For pristine WO_3_, this clearly occurs, expressed in the large reduction oxidation peaks. The WO_3_ film clearly leads to electron-transfer, facilitating a ferrocene reduction. While comparing this with the LLT-coated WO_3_, no reduction of the ferrocene can be observed. This implies that no surface is available for electron transfer although the ferrocene is fully exposed to the LLT surface. Hence, the lack of electron transfer indicates that the LLT electrolyte film is strongly electron insulating, which is crucial for application in an all-solid-state Li-ion battery.

**Figure 5:**
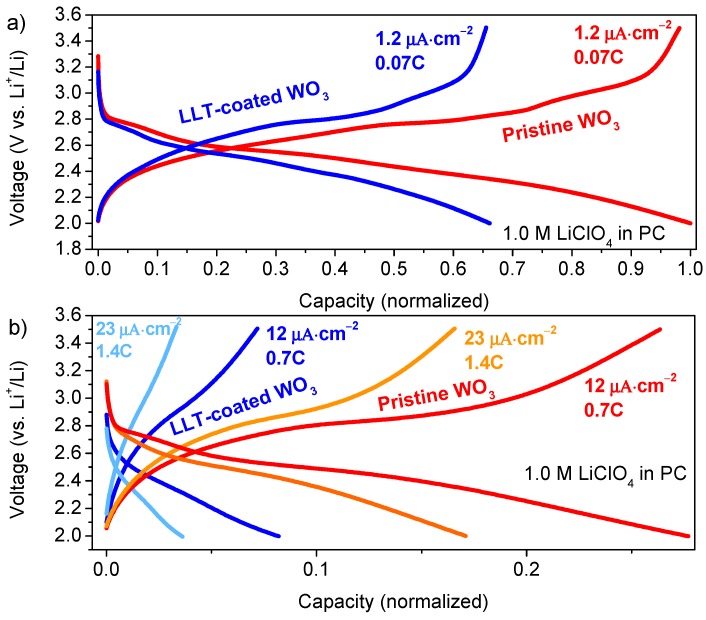
Lithiation/delithiation curves of 10 cycles W-precursor deposition on a planar Pt substrate, annealed at 600 °C for 1 h in static air and LLT-coated WO_3_ (prepared under the same conditions) annealed at 500 °C for 1 h in static air. The figure shows samples measured at: (**a**) low; and (**b**) high current densities. The capacity is normalized according to pristine WO_3_ cycled at 1.2 μA·cm^−2^ (0.07C). In all cases, the 10th cycle at the specified C-rate is shown.

Galvanostatic lithiation/delithiation curves of the deposited WO_3_, as shown in Figure 5, exhibit typical behavior observed for WO_3_ [33,34,35]; a short first plateau (2.7 V) followed by a longer plateau (2.6 to 2.4 V). Both plateaus are associated with the intercalation of Li^+^ in the tetragonal and cubic phase, respectively [35,53]. As was expected based on the CV results (Figure 3a), kinetic factors and/or amorphous phases appear to be present and responsible for intercalation at lower voltages, expressed in long slopes instead of charge plateaus [25]. Since the capacity drops to lower values at high current densities (Figure 5b), kinetic factors seem the most plausible reason for the occurrence of these slopes.

At a first glance, the lithiation capacity of 1405 mAh·cm^−3^ (197 mAh·g^−1^) for the films measured at low current densities of 1.2 μA·cm^−2^ (0.07C) (Figure 5a) appeared to be very high (232% of WO_3_ theoretical capacity), especially compared to films measured with the 10-fold current density of 12 μA·cm^−2^ (0.7C) (Figure 5b) yielding 492 mAh·cm^−3^ (68 mAh·g^−1^, 81% of WO_3_ theoretical capacity). However, a closer inspection of samples cycled at high and low current densities showed a striking macroscopic difference. As an electrochromic material, the lithiation of WO_3_ is macroscopically visible on the film due to dark blue coloration of reduced tungsten [52]. After a single lithiation down to 2.0 V, with a high current density of 12 μA·cm^−2^ (0.7C), a dark blue coloration of the sample measured was confined within the O-ring of the electrochemical cell (Figure S2a), as was expected. However, at a low current density of 1.2 μA·cm^−2^ (0.07C), the dark blue coloration was visible over all the substrate, including the area outside of the O-ring. XRD of selected areas (Figure S2b) indicated the same, as the cubic WO_3_ (implying lithiation occurred) was observed outside the O-ring area for low current densities. Since electron transfer of the Pt to the WO_3_ is possible all over the substrate, it is speculated that the relative high Li-ion diffusion through the WO_3_ film is responsible for this.

With diffusion constants of lithium in (cubic) WO_3_ in the order of 10^−8^ to 10^−7^ cm^2^·s^−1^ [22,55], a simple calculation (stochastic diffusion without convection) indicates that an average diffusion length in the millimeter range is feasible within the timescale of the experiment. However, unknown effects caused by (long) exposure to the liquid electrolyte are not excluded, but more detailed analysis of this topic goes beyond the scope of this study. Hence, the total capacity of the planar films reported in this study are normalized based capacity measured at the lowest current density, since exact determination of the capacity at low current densities is impossible without the confined volume approximation. The lithiation/delithiation curves of the LLT-coated WO_3_ (Figure 5) exhibit similar features as the pristine WO_3_—including the charge plateaus of the WO_3_ phases, although total capacity is significantly smaller. The capacity reduction can partly be ascribed to the presence of the inactive Li_2_W_2_O_7_ phase, as was shown by XRD (Figure 3b). However, the capacity reduction can also be related to a decreased rate performance induced by the LLT layer, as was already shown by the fact that a larger over-potential and smaller peak current were measured during CV (Figure 3a). The lack of confined volume explained also applies here at low current densities, but the effect is probably less severe because of the resistive character of the LLT electrolyte on top. Although the confined volume approximation applies at higher current density for the LLT coated samples, a larger capacity reduction is observed compared to pristine WO_3_ (Figure 5b). Thus, the smooth morphology and electronic blocking ensure crucial functions of the LLT electrolyte, but the amorphous character comes at a cost: reduced rate performance of the half-cell.

### 3.2. Tungsten Oxide and Lithium Lanthanum Titanium Oxide Stacks 3D Deposition

**Figure 6:**
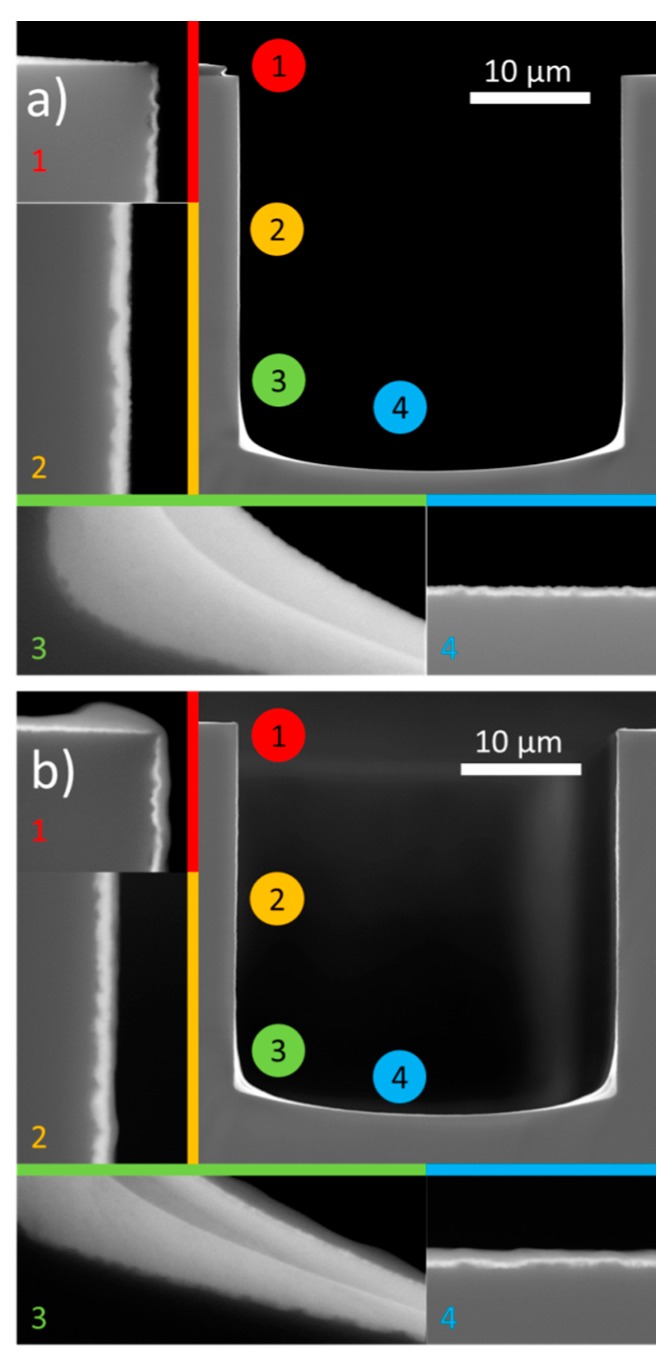
(**a**) SEM micrograph of 10 cycles of W-precursor deposition on a trench of 30 by 30 μm, annealed at 500 °C for 10 min in static air; and (**b**) additional deposition of 10 layers of Li-La-Ti-precursor deposition at 200 °C with an anneal at 500 °C for 10 min. Numbers indicate enlarged image locations.

Now that activity of both materials has been shown, the next step is taken towards forming an integrated 3D all-solid-state Li-ion battery: the non-planar deposition of WO_3_-LLT stacks. To study this extraordinary deposition requirement, Si substrates with trenches were used which were approximately 30 μm deep and 30 μm wide, i.e., aspect ratio 1 [8,11]. Upon deposition of the W-precursor at the optimized deposition temperature of 180 °C [21], the heavy tungsten is clearly visible all over the trench using backscattered imaging (Figure 6a), i.e., a fully covering WO_3_ film is formed. The coating is thicker in the corners, which can be explained by a capillary pressure gradient, leading to enhanced flow towards the corners of the trench geometry [56].

However, the exact layer thickness quantification for the bottom corner is relatively difficult because of a possible reaction between the Si substrate and the tungsten oxide layer (forming, e.g., tungsten silicates) [57] at the thickest part of the deposited layer. This is observed in a local difference in tungsten concentration as expressed in intensity difference of the backscattered image. Similar results were found for the deposition of a W-precursor on a planar Si substrate (Figure S3a), while this phenomenon did not occur in presence of a buffer layer such as Pt (Figure 2). We therefore quantify, within the uncertainty explained above, that the conformality is only 2%, i.e., the minimum layer thickness (top edge of the trench) divided by the maximum layer thickness (bottom corner). Further increasing the aspect ratio of the trenches, i.e., decreasing the width of the trench, no longer allows a fully covering deposition (cf. Figure S3a,b). It is speculated that three main phenomena are causing the difference in conformality with respect to the high aspect ratio trenches: (i) available surface; (ii) thermophoretic forces; and (iii) capillary forces and surface tension.

(i) The probability of a droplet reaching the bottom of the trenches scales with the area of the trench opening (top). As the width of the trench reduces, less material is able to reach and spread at the bottom of the trench.
(ii) Thermophoretic forces, i.e., the repelling force caused by a huge pressure gradient near to the hot surface [58], are larger in the case of small trenches. Carrier gas diffusion into a small trench is more difficult than in to a broad trench, leading to an increased thermophoretic force for smaller trench. Therefore, layer deposition into deeper parts of the trenches is becoming more difficult.
(iii) A necking mechanism is observed, especially for the smallest type of trench measured. The surface tension of the gel leads to necking (contacting similar material) instead of wetting the alien (Si) material down the trench.

In view of materials stacking, only the trench with aspect ratio 1, coated with a fully covering WO_3_ coating, was used for the LLT-precursor deposition. The result in Figure 5b shows that a conformal LLT solid-electrolyte layer is deposited on top of the WO_3_ layer. In addition, the quantified conformality (33%) of the LLT layer is much better than for the WO_3_ deposition (2%), as the difference in thickness at the edge and bottom are considerably smaller. Besides possible differences in the surface chemistry, the large difference is probably generated by the fact that the sharp edge present in the uncoated trench is no longer present after coating with WO_3_. Hence, the LLT layer covers a curved surface instead of a sharp corner, which is beneficial for the deposition homogeneity. Although there is room for optimization of layer thicknesses, these results indicate that stacked fully covering coatings are possible using a wet-chemical approach.

### 3.3. Towards Functional WO_3_ 3D Coatings

#### 3.3.1. WO_3_ Deposition on High Aspect Ratio Substrates

The activity of the materials was demonstrated for planar substrates, in addition to full coverage of the non-planar trench structures with WO_3_. However, the capacity gain due to the use of 3D architectures is yet to be shown using this approach [3]. In order to do so, a substrate with a high aspect ratio and a suitable current collector was required. For this study, TiN coated micro-cylinders of 50 μm height, approximately 1 μm radius and inter-cylinder distance of 5 μm were available, enabling a potential 8-fold capacity increase. Figure S4 shows that good coatings could be obtained while submitting these micro-cylinders to the W-precursor spray, with an aspect ratio of 10. Although only limited aspect ratios were achieved for the trenches—with a “closed” structure (Figure 6a)—apparently higher aspect ratio “open” micro-cylinder can indeed be coated with this approach (Figure S4). It is speculated that the large openings (at the top) and beneficial capillary forces of these micro-cylinder structures attribute to this difference. In addition, no material accumulates at the top of the substrate, preventing the “necking” occurring in case of the trenches (Figure S3c). Besides SEM micrographs (Figure S4), the galvanostatic measurements also indicate the micro-cylinders are coated (cf. Section 3.3.3). Unlike the trench structures (Figure 5), cross sections by SEM were not possible on these micro-cylinders. ICP-AES based thickness estimations for the coated TiN/Si micro-cylinders indicate that the average WO_3_ film thickness for 16 deposition cycles is 32 nm.

#### 3.3.2. Thermal Decomposition and Crystallization

**Figure 7:**
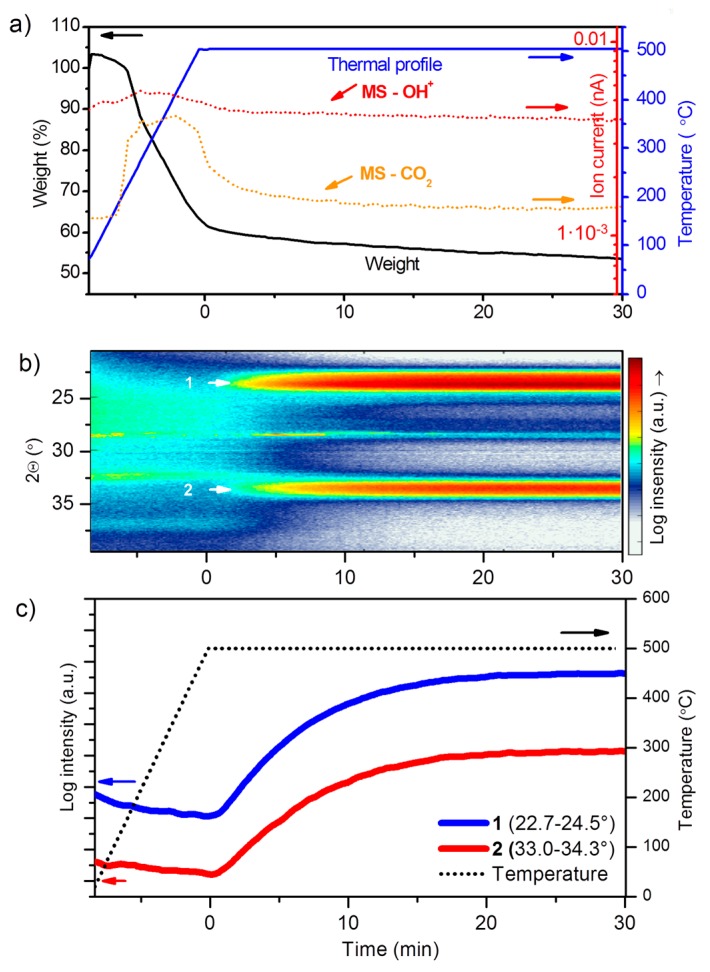
Analysis of as-deposited W-precursor films on: (**a**) glass showing TGA coupled with MS (10 °C·min^−1^ in static air, *m*/*z* 17/OH and 44/CO_2_) and isXRD on coated TiN/Si micro-cylinders showing: (**b**) peak intensity as function of peak position and time; and (**c**) integrated peak intensities of specified diffraction peaks as function of temperature, where blue is related to peak 1 and red to peak 2 to in (**b**).

Although TiN (present on the micro-cylinders) is a good electronic conductor and is very interesting for this specific application because of its excellent (Li) barrier properties [59], the TiN current collector is highly susceptible to oxidation at elevated temperatures in air, which leads to the formation of TiO_2_ with a low electronic conductivity [60]. This therefore initially resulted in unmeasurable samples (not shown). Optimized annealing conditions were therefore sought, in order to find an optimum between precursor decomposition, crystallization and electrochemical activity of the material (WO_3_) on the one hand, and preservation of the current collector (TiN) on the other. Isothermal heating at 500 °C was applied to decompose the W-citrate, preserving the TiN current collector for a limited time. The non-isothermal TGA profile of the W-precursor suggests that part of the citrate complex is not fully decomposed at 500 °C, even while applying an isotherm at this temperature (not shown). To make sure that heat transfer, volume and geometry differences concerning the decomposition analysis with in-situ XRD (isXRD) and TGA were minimized, deposited films instead of powders were measured [61]. The result is a weight loss profile (Figure 7a) showing continuous weight loss, but to a smaller extent than in the case of gels (not shown), which is mostly attributed to higher heat transfer rates and a larger surface for gas evolution for film-based TGA. Using coupled MS methods, the weight loss could be assigned to CO_2_ and H_2_O, meaning that a continuous combustion of organic groups occurs during the isothermal period at 500 °C. The slow combustion occurs together with a gradual increase of crystallinity, which was monitored in real-time using isXRD (Figure 7b,c). Since W–O–W bonds are required for crystallization of WO_3_, W–O–C bonds of the citrate complex should be broken. The gradual but immediate crystallization was to be expected, as amorphous WO_3_ is reported to crystalize at temperatures as low as 300 °C [24,36]. However, this does not fully exclude occurrence of decomposing residual organic groups after crystallization, since ammonium citrate (partly decomposed) residues or detached citrate ligand groups can decompose after crystallization. The crystallization increases over time to form the tetragonal phase of WO_3_.

A relatively large gain in intensity is found during the first 10 min of the isothermal anneal as approximately 75% of the maximum intensity is reached within this time for the 23.5 and 33.5 peaks (Figure 7c). This matches with the H_2_O and CO_2_ evolution, which show a decreasing intensity over this period of time. Therefore, 10 min of isothermal heating was chosen as an optimum between film crystallization and preservation of the current collector (TiN) present on the micro-cylinders.

#### 3.3.3. Functional Measurements of 3D WO_3_ Coatings

**Figure 8:**
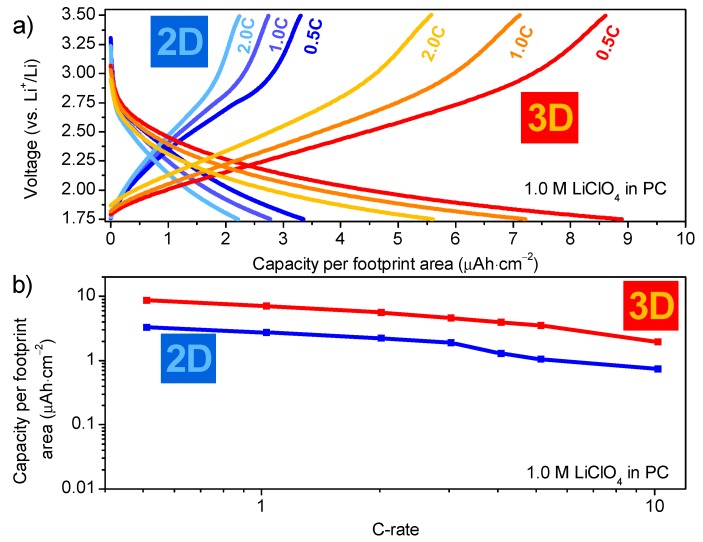
(**a**) Lithiation/delithiation curves; and (**b**) delithiation capacity-rate log-log plot of 2D (Pt) and 3D (TiN) WO_3_ coated substrates, annealed at 500 °C for 10 min in static air. 2D samples were subjected to two deposition cycles, and 3D sampled (with a factor 8 area enhancement) were subjected to 16 deposition cycles. In all cases, data of the 10th cycles are shown and volumetric capacities are determined with ICP-based (2D and 3D) coating thicknesses.

Despite the presence of residual organic fragments and incomplete crystallization after only 10 min of isothermal heating at 500 °C, active WO_3_ present on the micro-cylinders could be cycled as shown in Figure 8. To study the effect of 3D deposition more thoroughly than earlier reports by our labs [21], the micro-cylinder sample was compared with a planar substrate exhibiting the same amount of material per footprint area (i.e., 2 deposition cycles for 2D, compared to 16 deposition cycles for 3D with an area enhancement factor of 8). In addition, the current applied to the 3D samples is eight fold the current passed through the 2D samples (Figure 8). The lithiation/delithiation curves look similar for 2D and 3D, although the capacity is significantly larger for the 3D sample. All curves in Figure 8a lack plateaus as observed on planar samples which were annealed longer at higher temperature. Instead, slopes are observed, especially during delithiation. This is an indicator that primarily amorphous WO_3_ is present in the samples [25], despite indications of isXRD that part of the material is crystalline (Figure 7b). Since these diffraction peaks are broad compared to a non-isothermal anneal at higher temperature (Figure S5), it can be concluded that the crystallites are rather small, i.e., occurrence of nano-crystallinity. It is speculated that these nanocrystals might lead to comparable, (quasi) amorphous lithiation/delithiation behavior. At 0.5C, the two 2D sample is able to store a charge of 3.35 μAh·cm^−2^, whereas the 3D sample stores 8.90 μAh·cm^−2^ with a comparable current per equivalent coating area. This is a 2.6-fold capacity improvement which can be attributed to the benefit that 3D geometries exhibit over 2D electrodes. This result equals the capacity enhancement obtained for LTO deposited using CVD, on similar high aspect ratio substrates (TiN coated micro-cylinders) which achieved a 2.5 capacity enhancement as well as previous reports from our labs reaching a near three fold capacity increase [8,21]. However, these results were achieved by applying the equal current density per footprint area, whereas the current study shows that a more critical equal current per coating area. In addition, it enables to study the kinetic performance of the 3D structured films in more detail (Figure 8b). Nevertheless, the eight-fold capacity enhancement, which theoretically should be achievable, is not met. There are several reasons for not achieving the theoretical capacity increase:

(i) The deposition of W-citrate on the trench structures (Figure 6a) illustrates that layer thickness cannot be controlled perfectly, which leads to local differences in capacity of the layer, therefore lowering the capacity enhancement.
(ii) Although the coating is present on the micro-cylinders, certain defects can be observed (Figure S4).
(iii) The current collector may remain an issue; upon cycling, the voltage drop at the start of the measurement is significantly larger for the 3D samples than for the 2D samples (Figure 8a). This illustrates that higher resistance is presence in these 3D samples, which is primarily attributed to the partial oxidation of the TiN current collector (20 nm). As the TiN current collector is much thicker for the 2D samples (80 nm), and the applied currents are much lower, this leads to lower IR-drop for planar films, compared to the 3D counterparts.

Nevertheless, the de-lithiation kinetics of the 2D and 3D film are comparable between 0.5C and 10C (Figure 8b). The same slope of the lines in the double log plot indicate the same kinetic regime acts on both films [11], where the 2.6 fold capacity enhancement is maintained up to high current densities.

## 4. Conclusions

All things considered, the stacking of lithium lanthanum titanate (LLT) with tungsten oxide (WO_3_) yielded an active all-solid-state half-cell, showing both the Li-ion conducting and electron blocking function of the electrolyte layer. With smooth morphologies and relatively mild thermal budgets (500 °C), the combination of citrate-nitrate chemistry led to active 3D structured materials within the thermal budget limitations. This is another step forward within the field of wet-chemical synthesis, since 3D depositions were previously limited to all-citrate based precursors. This adds to the understanding of this complicated, fascinating process to establish high aspect ratio oxide coatings. Although additional progress is required to suppress secondary phase formation and enhancement of the Li-ion conductivity of the half-cell, the results of this study form the beginning of tackling problems related to the Achilles’ heel of the all-solid-state 3D Li-ion battery research: 3D deposition of suitable solid-electrolyte materials. Besides the advancements made for the solid-electrolyte, the near threefold capacity enhancement of the 3D-structured WO_3_ negative electrode is an important indicator of the versatility of wet-chemical method in combination with ultrasonic spray deposition. Future improvements should be booked to obtain a suitable current collector with a larger thermal budget, as this is the limiting factor while probing the performance of 3D-structured electrode materials. Nevertheless, stacking of oxide materials using ultrasonic spray deposition—in 3D—is a major step forward in terms of possible new material architectures, especially because both layers were deposited with an easy-to-upscale process that can possibly be applied to many other applications as well.

## Supplementary Materials

The following are available online at www.mdpi.com/1996-1944/10/9/1072/s1, Figure S1: TGA analysis of (a) the lithium nitrate precursor and (b) lanthanum nitrate precursor dried at 60 °C, recorded at 10 °C·min^−1^ in dry air (0.1 ml·min^−1^), Figure S2: Comparison between WO_3_ coated (10 cycles, 25 mM) Pt samples after a single lithiation down to 2.0 V vs Li^+^/Li, at high and low current density. (a) Photograph of the samples and O-ring used in the electrochemical cell, showing dark blue coloration of lithiated areas. The colored squares relate to (b), showing XRD on isolated parts after cutting the samples, Figure S3: SEM micrograph showing backscattered (BSE) image of 10 cycles of W-precursor deposition on a (a) planar Si substrate, (b) trench of 10 by 27 μm and (c) trench of 3.5 by 22.5 μm, all annealed at 500 °C for 10 min in static air, Figure S4: SEM micrograph of 10 cycles of W-precursor deposition at 180 °C on 50 μm high micro-cylinders, with an average diameter of 2.5 μm, with 5 μm inter-cylinder spacing. The sample was annealed at 500 °C for 10 min in static air, Figure S5: In-situ XRD results showing applied temperature profile (top), as well as (bottom) diffraction intensity as function of peak position and time. Both graphs are based on the same sample; W-citrate deposited using 10 cycles on TiN micro-cylinders at a deposition temperature of 180 °C.
