# Supplementary material for: Wet-Chemical Synthesis of 3D Stacked Thin Film Metal-Oxides for All-Solid-State Li-Ion Batteries
Source: Materials (Basel). 2017 Sep 12;10(9):1072. doi: 10.3390/ma10091072 (PMC5615726; doi:10.3390/ma10091072)
Supplement: Supplementary file 1 [file materials-10-01072-s001.pdf]

# Supplementary Materials

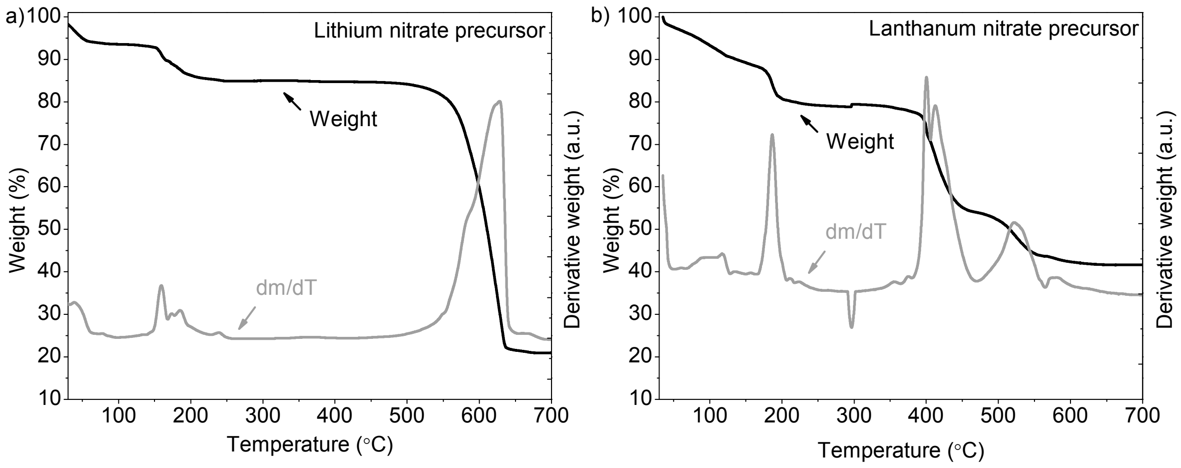


**Figure S1.** TGA analysis of (**a**) the lithium nitrate precursor and (**b**) lanthanum nitrate precursor dried at 60 °C, recorded at 10 °C·min$^{-1}$ in dry air (0.1 ml·min$^{-1}$).

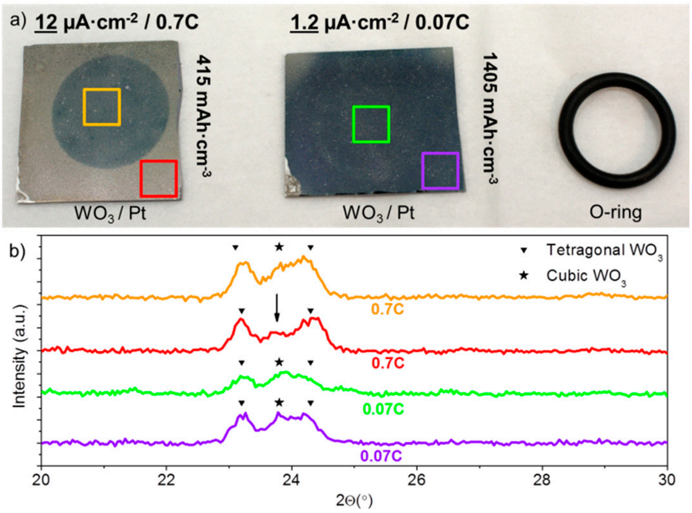


**Figure S2.** Comparison between $WO_3$ coated (10 cycles, 25 mM) Pt samples after a single lithiation down to 2.0 V vs $Li^+$/Li, at high and low current density. (**a**) Photograph of the samples and O-ring used in the electrochemical cell, showing dark blue coloration of lithiated areas. The colored squares relate to (**b**), showing XRD on isolated parts after cutting the samples.

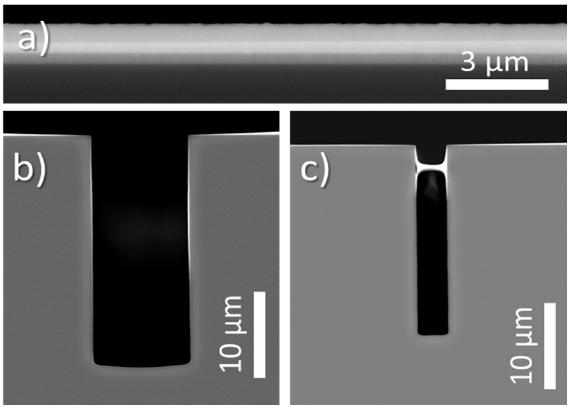


**Figure S3.** SEM micrograph showing backscattered (BSE) image of 10 cycles of W-precursor deposition on a (**a**) planar Si substrate, (**b**) trench of 10 by 27 μm and (**c**) trench of 3.5 by 22.5 μm, all annealed at 500 °C for 10 min in static air.

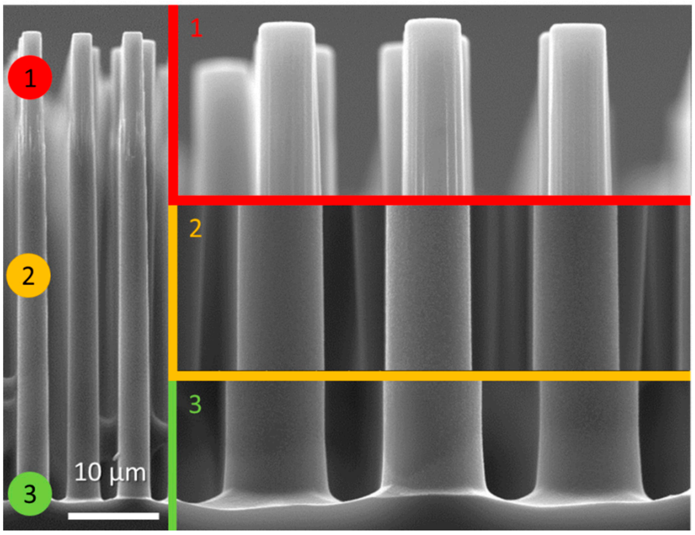


**Figure S4.** SEM micrograph of 10 cycles of W-precursor deposition at 180 °C on 50 µm high micro-cylinders, with an average diameter of 2.5 µm, with 5 µm inter-cylinder spacing. The sample was annealed at 500 °C for 10 min in static air.

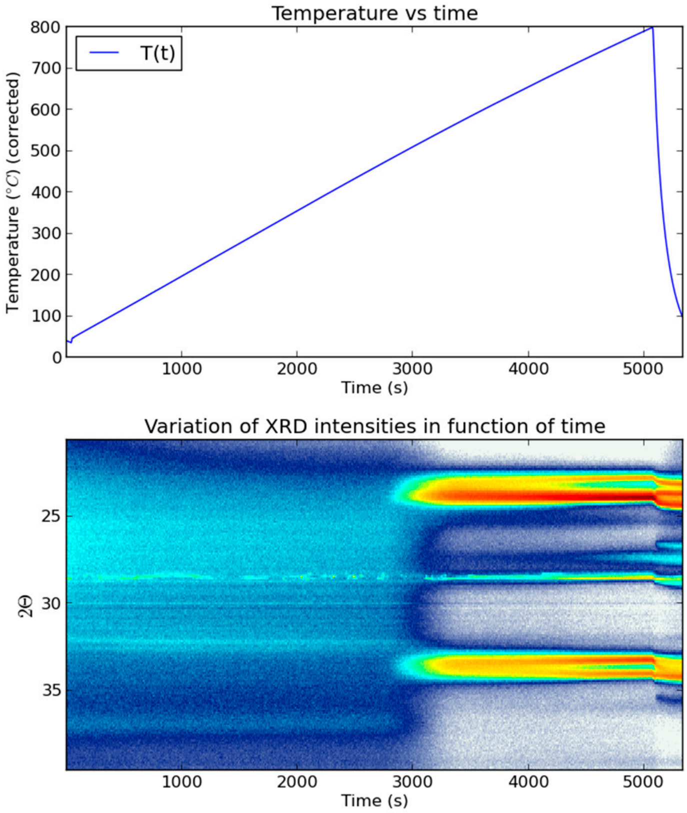


**Figure S5.** In-situ XRD results showing applied temperature profile (top), as well as (bottom) diffraction intensity as function of peak position and time. Both graphs are based on the same sample; W-citrate deposited using 10 cycles on TiN micro-cylinders at a deposition temperature of 180 °C.
